# Thrombocytopenia in Anti-neutrophil Cytoplasmic Antibody-Associated Vasculitis Indicating the Presence of Cytomegalovirus Infection: A Case Report

**DOI:** 10.7759/cureus.38850

**Published:** 2023-05-10

**Authors:** Ryuichi Ohta, Yumi Naito, Nozomi Nishikura, Keita Inoue, Chiaki Sano

**Affiliations:** 1 Community Care, Unnan City Hospital, Unnan, JPN; 2 Urology, Unnan City Hospital, Unnan, JPN; 3 Community Medicine Management, Shimane University Faculty of Medicine, Izumo, JPN

**Keywords:** general medicine, japan, rural hospital, older patients, intracytoplasmic inclusion body, thrombocytopenia, anti-neutrophil cytoplasmic antibody, cytomegalovirus infection

## Abstract

Although cytomegalovirus (CMV) usually colonizes the human body without causing symptoms, CMV infections often develop in immunocompromised hosts. Immunosuppression can trigger CMV infection, and its prediction is essential; however, this is challenging without specific criteria.

We present the case of an 87-year-old male patient who visited a rural community hospital with the chief complaint of persistent cough, productive of bloody sputum. Initially, the patient developed thrombocytopenia without any abnormalities of liver function; however, a positive myeloperoxidase antineutrophil cytoplasmic antibody (ANCA) test and the presence of alveolar hemorrhage and glomerulonephritis confirmed ANCA-associated vasculitis. The patient’s symptoms and thrombocytopenia resolved transiently after treatment with prednisolone and rituximab. However, the recurrence of thrombocytopenia and the appearance of urinary intracytoplasmic inclusion bodies during the treatment course were investigated using an antigenemia test, which ultimately confirmed CMV viremia. Valganciclovir treatment resolved all the symptoms. This case report showed that thrombocytopenia might indicate the presence of CMV infection in ANCA-associated vasculitis and that intracytoplasmic inclusion bodies in immunosuppressed patients require investigation of CMV infection for effective treatment.

## Introduction

Although cytomegalovirus (CMV) usually colonizes the human body without causing symptoms, CMV infections often develop in immunocompromised hosts. Generally, 50-80% of adults would have been infected with CMV. By age 40, they have CMV infection [1]. However, in most cases, CMV does not cause symptoms in immunocompetent patients, in whom proliferation is suppressed. In primary care settings, patients with CMV infections do not require intensive care [2].

Predicting the development of CMV infection in older immunocompetent patients is essential, but it can be challenging due to the triggering effect of immunosuppression [3,4]. The symptoms of CMV infection vary, with most being vague, and include mild fever, fatigue, sore throat, muscle aches, enlarged lymph nodes, headache, and appetite loss [5]. Laboratory abnormalities have been observed in patients with advanced cases of CMV infections [5]. Investigation of CMV in immunosuppressed patients with vague symptoms is mandatory [5]. However, investigations in older patients without an immunosuppressed clinical background are controversial.

We report the case of an 87-year-old male patient who visited a rural community hospital with the chief complaint of persistent cough productive of bloody sputum. When the patient first began to develop thrombocytopenia without any liver function abnormalities, however, a positive test result for myeloperoxidase antineutrophil cytoplasmic antibody (ANCA) and the presence of alveolar hemorrhage and glomerulonephritis led to the diagnosis of ANCA-associated vasculitis. The patient’s symptoms and thrombocytopenia resolved transiently after treatment with prednisolone and rituximab. However, the recurrence of thrombocytopenia and the appearance of urinary intracytoplasmic inclusion bodies during the treatment course were investigated using an antigenemia test, which eventually confirmed CMV viremia. In this case report, we discuss the importance of thrombocytopenia and intracellular inclusion bodies in investigating CMV infections.

## Case presentation

An 87-year-old male patient presented at a rural community hospital with a one-week history of persistent cough, productive of bloody sputum. The patient had a dry cough without bloody sputum one month before presentation; however, two weeks before admission, he noticed blood-containing sputum. His cough worsened one week before admission, and the amount of bloody sputum increased. On the day of admission, he had a mild fever, could not sleep, and had a persistent cough; therefore, he was admitted to our hospital. He lived in a nursing home with no recent travel history or contact with an infected person. The patient had no history of trauma, chills, or other symptoms of infection. His medical background comprised hypertension, dyslipidemia, and bilateral knee osteoarthritis. His medication history included amlodipine (5 mg/day) and acetaminophen (500 mg/day).

The patient’s vital signs on presentation were as follows: blood pressure, 89/52 mmHg; pulse, 109 beats/min; body temperature, 37.1 °C; respiratory rate, 24 breaths/min; and oxygen saturation, 98% on room air. The patient was alert and oriented in time, place, and person. Physical examination revealed bilateral late inspiratory crackles on the chest, without heart murmurs or limb edema. No neurological abnormalities were observed. No abdominal abnormalities or skin eruptions are observed. The laboratory testing results demonstrated severe inflammation with thrombocytopenia, hematuria, and proteinuria (Table 1).

**Table 1:** Initial laboratory data of the patient eGFR, estimated glomerular filtration rate; CK, creatine kinase; CRP, C-reactive protein; TSH, thyroid-stimulating hormone; Ig, immunoglobulin; HCV, hepatitis C virus; SARS-CoV-2, severe acute respiratory syndrome coronavirus 2; HIV, human immunodeficiency virus; HBs, hepatitis B surface antigen; HBc, hepatitis B core antigen; C3, complement component 3; C4, complement component 4; MPO-ANCA, myeloperoxidase anti-neutrophil cytoplasmic antibody

Parameter	Level	Reference
White blood cells	10.1	3.5–9.1 × 10^3^/μL
Neutrophils	87.9	44.0–72.0%
Lymphocytes	3.9	18.0–59.0%
Monocytes	8.0	0.0–12.0%
Eosinophils	0.1	0.0–10.0%
Basophils	0.1	0.0–3.0%
Red blood cells	3.24	3.76–5.50 × 10^6^/μL
Hemoglobin	9.4	11.3–15.2 g/dL
Hematocrit	28.2	33.4–44.9%
Mean corpuscular volume	87.0	79.0–100.0 fL
Platelets	6.6	13.0–36.9 × 10^4^/μL
Erythrocyte sedimentation rate	50	2–10 mm/hour
Total protein	5.8	6.5–8.3 g/dL
Albumin	2.5	3.8–5.3 g/dL
Total bilirubin	0.9	0.2–1.2 mg/dL
Aspartate aminotransferase	24	8–38 IU/L
Alanine aminotransferase	12	4–43 IU/L
Alkaline phosphatase	71	106–322 U/L
γ-Glutamyl transpeptidase	16	<48 IU/L
Lactate dehydrogenase	167	121–245 U/L
Blood urea nitrogen	23.4	8–20 mg/dL
Creatinine	0.60	0.40–1.10 mg/dL
eGFR	90	>60.0 mL/min/L
Serum Na^+^	133	135–150 mEq/L
Serum K^+^	3.7	3.5–5.3 mEq/L
Serum Cl^+^	98	98–110 mEq/L
Serum Ca^2+^	8.3	8.8–10.2 mg/dL
Ferritin	99.0	14.4–303.7 ng/mL
CK	75	56–244 U/L
CRP	21.84	<0.30 mg/dL
TSH	1.27	0.35–4.94 μIU/mL
Free T4	1.38	0.70–1.48 ng/dL
IgG	1456	870–1700 mg/dL
IgM	59	35–220 mg/dL
IgA	368	110–410 mg/dL
IgE	195	<173 mg/dL
HBs antigen	0.0	IU/mL
HBs antibody	0.67	mIU/mL
HCV antibody	0.00	S/CO
Syphilis treponema antibody	0.00	S/CO
SARS-CoV-2 antigen	-	
anti-nuclear antibody	40	<40
C3	75	86–164 mg/dL
C4	18	17–45 mg/dL
MPO-ANCA	88.3	<3.5 U/mL
Urine test		
Leukocyte	(1+)	Negative
Nitrite	Negative	Negative
Protein	(1+)	Negative
Glucose	Negative	Negative
Urobilinogen	Negative	
Bilirubin	Negative	Negative
Ketone	Negative	Negative
Blood	(2+)	Negative
pH	5.5	
Specific gravity	1.022	

**Figure 1:**
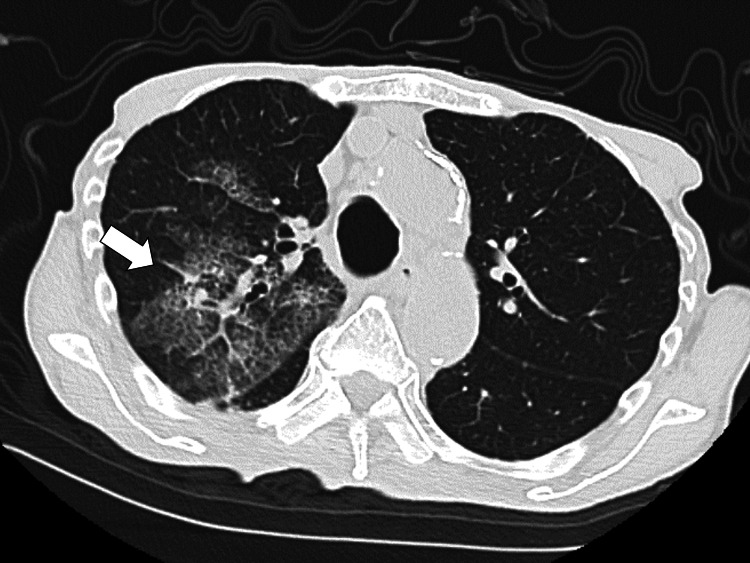
Computed tomography of the chest showing multiple lesions of ground-glass opacities on bilateral lungs showing alveolar hemorrhage (white arrow)

Chest radiography revealed bilateral infiltration in the upper parts of both lungs. Chest computed tomography (CT) revealed multiple lesions with grand glass opacities in both lungs, indicating alveolar hemorrhage (Figure 1).

On day 3 of admission, additional laboratory tests revealed a myeloperoxidase ANCA of 88.3 U/mL. On day 5 of admission, a renal biopsy revealed glomerulonephritis.

**Figure 2:**
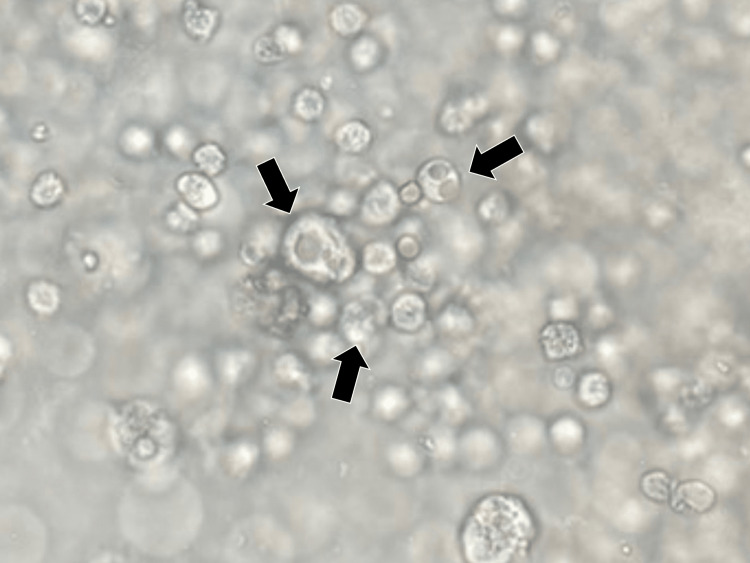
The urinalysis showing multiple intracytoplasmic inclusion bodies/high power fields (block arrows)

Initially, the patient developed thrombocytopenia without any abnormalities of liver function. Positive myeloperoxidase ANCA, suspected alveolar hemorrhage, and glomerulonephritis led to a diagnosis of ANCA-associated vasculitis. After treatment with prednisolone (40 mg) and rituximab (500 mg) on day 7 of admission, the hemoptysis and thrombocytopenia disappeared transiently. However, on day 28 of admission, after the third rituximab dose of 500 mg, the patient’s laboratory data showed a recurrence of thrombocytopenia (9.5×104/mL). Urinalysis performed on the same day revealed multiple intracytoplasmic inclusion bodies per high-power field (Figure 2).

Considering the thrombocytopenia and urinary intracytoplasmic inclusion bodies after the administration of rituximab, CMV infection was suspected. CMV antigenemia test confirmed CMV viremia. On day 31 of admission, oral valganciclovir (900 mg/day) was initiated. On day 36 of admission, the patient’s platelet count increased to 17.5×104/mL. On day 45 of admission, all the patient’s symptoms had resolved. The patient was discharged and followed up in the outpatient department.

## Discussion

This case report shows that thrombocytopenia may indicate the presence of CMV infection in ANCA-associated vasculitis and that intracytoplasmic inclusion bodies in immunosuppressed patients require investigation for CMV infection, leading to effective treatments.

Thrombocytopenia in older adults should be investigated within the framework of immunosuppressive therapy. Usually, bone suppression-induced thrombocytopenia can be caused by acute viral and bacterial infections due to highly inflammatory conditions [6]. Malignancies and autoimmune diseases rarely cause thrombocytopenia [6]. However, as in the present case, immunocompromised conditions may have altered the differential diagnosis of thrombocytopenia [7]. Various viral infections can cause occult viremia without subjective symptoms [8]. CMV infection, which is a viral infection [9], is prevalent among older asymptomatic patients, and most patients do not require treatment with antiviral drugs [10]. In contrast, thrombocytopenia may be an initial manifestation of active CMV infection [11]. To effectively treat older patients requiring immunosuppressive treatment, physicians should check for CMV viremia among patients with thrombocytopenia, and detect and treat active infections before treatment.

Urinary intracytoplasmic inclusion bodies can be used to detect CMV infection in immunosuppressed patients. In this case, the detection of urinary intracytoplasmic inclusion bodies was the starting point for the investigation of CMV infection. Urinary intracytoplasmic inclusion bodies can be found in various medical conditions, including bacterial and viral tract infections [12]. Intracytoplasmic inclusion bodies can be detected mainly in systemic viral infections caused by CMV, Epstein-Barr virus, and other herpes viruses [13,14]. Understanding the clinical course is critical for understanding the clinical relevance of cytoplasmic inclusion bodies. In this case, the patient did not have acute symptoms, such as fever or distress, suggesting acute infections. In contrast, platelet counts were lower in the absence of other symptoms, which could be the only symptom of CMV infection. A previous report showed that the frequency of low platelet count among CMV infections was 80% in older patients with systemic symptoms [3]. This case showed that only thrombocytopenia could be an initial clinical finding in CMV infections; therefore, clinicians should suspect CMV infection when investigating the etiology of thrombocytopenia.

## Conclusions

Thrombocytopenia may indicate CMV infection in older immunocompetent patients. In ANCA-associated vasculitis treatment, urinary intracytoplasmic inclusion bodies may indicate CMV infection exacerbation and should be investigated for effective treatment.
